# The Allergic Rhinitis – Clinical Investigator Collaborative (AR-CIC): nasal allergen challenge protocol optimization for studying AR pathophysiology and evaluating novel therapies

**DOI:** 10.1186/s13223-015-0082-0

**Published:** 2015-04-24

**Authors:** Anne K Ellis, Mena Soliman, Lisa Steacy, Marie-Ève Boulay, Louis-Philippe Boulet, Paul K Keith, Harissios Vliagoftis, Susan Waserman, Helen Neighbour

**Affiliations:** Departments of Medicine and Biomedical & Molecular Science, Queen’s University, Kingston, ON Canada; Allergy Research Unit, Kingston General Hospital, Kingston, ON Canada; Institut Universitaire de Cardiologie et de Pneumologie de Quebec, Quebec, QC Canada; Department of Medicine, McMaster University, Hamilton, ON Canada; Pulmonary Research Group, University of Alberta, Edmonton, AB Canada; Firestone Institute for Respiratory Health, McMaster University, Hamilton, ON Canada

**Keywords:** Allergic rhinitis, Nasal allergen challenge, Allergic rhinitis, Clinical investigator collaborative

## Abstract

**Background:**

The Nasal Allergen Challenge (NAC) model allows the study of Allergic Rhinitis (AR) pathophysiology and the proof of concept of novel therapies. The Allergic Rhinitis – Clinical Investigator Collaborative (AR-CIC) aims to optimize the protocol, ensuring reliability and repeatability of symptoms to better evaluate the therapies under investigation.

**Methods:**

20 AR participants were challenged, with 4-fold increments of their respective allergens every 15 minutes, to determine the qualifying allergen concentration (QAC) at which the Total Nasal Symptom Score (TNSS) of ≥10/12 OR a Peak Nasal Inspiratory Flow (PNIF) reduction of ≥50% from baseline was achieved. At the NAC visit, the QAC was used in a single challenge and TNSS and PNIF were recorded at baseline, 15 minutes, 30 minutes, 1 hour, and hourly up to 12 hours. 10 additional ragweed allergic participants were qualified at TNSS of ≥8/12 AND ≥50% PNIF reduction; the Cumulative Allergen Challenge (CAC) of all incremental doses was used during the NAC visit. 4 non-allergic participants were challenged with the highest allergen concentration.

**Results:**

In the QAC study, a group qualified by only meeting PNIF criteria achieved lower TNSS than those achieving either TNSS criteria or PNIIF+TNSS (p<0.01). During the NAC visit, participants in both studies reached their peak symptoms at 15minutes followed by a gradual decline, significantly different from non-allergic participants. The “PNIF only” group experienced significantly lower TNSS than the other groups during NAC visit. QAC and CAC participants did not reach the same peak TNSS during NAC that was achieved at screening. QAC participants qualifying based on TNSS or TNSS+PNIF managed to maintain PNIF scores.

**Conclusions:**

Participants experienced reliable symptoms of AR in both studies, using both TNSS and PNIF reduction as part of the qualifying criteria proved better for qualifying participants at screening. Phenotyping based on pattern of symptoms experienced is possible and allows the study of AR pathophysiology and can be applied in evaluation of efficacy of a novel medication. The AR-CIC aims to continue to improve the model and employ it in phase 2 and 3 clinical trials.

## Background

Allergic rhinitis (AR) is an upper airway inflammatory disease, characterized by symptoms of rhinorrhea, sneezing, and nasal congestion. In addition, non-nasal symptoms such as itching of the throat and/or palate, and conjunctival symptoms can occur in individuals with AR with exposure to their relevant allergen(s). Although AR is often considered a benign condition, it greatly impacts the quality of life of affected individuals, with economic costs estimated at $5.3 billion per year in the USA [1,2]. Worldwide prevalence of AR is estimated at 9% to 42% [3,4], however it is hard to quantify due to inconsistency of population sampling methods that are employed in different countries. There are also challenges when trying to assess the efficacy of new medications in the treatment of AR. Day-to-day variability in outdoor allergen concentration, effects of weather including temperature and humidity, as well as participant’s lifestyle, can all potentially affect nasal and ocular symptom scores, common outcomes in these studies [5,6].

A standardized and efficient model which controls many environmental variables is essential to reliably evaluate the efficacy of new medications in the treatment of AR. One such model, the Environmental Exposure Unit (EEU), is a model of human allergen exposure, where allergen exposure is controlled, and patients can be continuously monitored for symptoms [7,8]. EEUs are effective and provide reproducible and reliable results, but require special infrastructure and expertise that is not commonly available at most sites [5]. The model, however, does not allow for individual titration of the allergen and biological sampling (e.g. nasal lavage) from a large number of participants can be challenging.

Nasal Allergen Challenge (NAC) is another human model of AR that involves the direct exposure of the nasal mucosa to allergen. The NAC has the advantage of running pilot studies with a small number of participants as well as allowing biological sampling. The inflammatory response that ensues, including early and late phase reactions (in a subset of participants), is similar to that observed in people with symptoms of AR during natural exposure [9-11]. Disease pathophysiology and the effect of medications can be studied through monitoring cytokines and inflammatory cells, allowing for accurate measurements of drug efficacy and onset/duration of action for each group of participants experiencing early and/or late phase responses as applicable to the mechanism of action [12]. The NAC model has proven reliable through many clinical trials of anti-histamines [13-16], intranasal corticosteroids [17-20], and subcutaneous and sublingual immunotherapies [21-24], albeit through variable methodologies.

To address these issues, the Allergic Rhinitis – Clinical Investigator Collaborative (AR-CIC) was formed with funding from the AllerGen Network of Centres of Excellence (AllerGenNCE). The AR-CIC is a Canadian multi-center initiative with the primary goal of conducting standardized NAC protocols to study the pathophysiology of AR as well as the mechanism of action and therapeutic effects of novel therapies. A reliable NAC model, would provide “a proof of principle” that a novel medication may be effective, prior to proceeding to a clinical trial. The NAC model was chosen as it is possible to test participants at multiple sites in controlled conditions and outside of their respective pollen season, where changes in symptoms or biological markers can be directly attributed to the NAC and study medication [25,26]. The allergen dose administered can be customized to the individual patient, allowing all participants to achieve the required level of AR symptoms.

Researchers have used a variety of methods to introduce allergen into the nasal cavity, which have been described elsewhere [6,25-34]. Several methods are available for measurement of the nasal symptoms experienced by study participants. The Total Nasal Symptom Score (TNSS) is the sum of scores for each of nasal congestion, sneezing, nasal itching, and rhinorrhea at each time point, using a four point scale (0–3), where 0 indicates no symptoms, a score of 1 for mild symptoms that are easily tolerated, 2 for awareness of symptoms which are bothersome but tolerable and 3 is reserved for severe symptoms that are hard to tolerate and interfere with daily activity (Table 1). TNSS is calculated by adding the score for each of the symptoms to a total out of 12. Another method, the Visual Analogue Scale (VAS), is a 10 cm scale that ranges from “no symptoms” to “worst symptoms ever” for each of the nasal symptoms [35]. Both methods are subjective, and thus depend on the tolerance and perceptions of the participant, which may cause variability in the data [36].

**Table 1:** **Total Nasal Symptom Scores (TNSS) Each symptom (sneezing, congestion, itching, and rhinorrhea) is graded from 0-3 by the participants during the screening and NAC visits**

**Score**	**Symptoms**
0=None	No symptoms evident
1=Mild	Symptom present but easily tolerated
2=Moderate	Definite awareness of symptom; bothersome but tolerable
3=Severe	Symptom hard to tolerate; interferes with daily activity

The Nasal symptoms are then added for each time points to reach the TNSS.

Peak Nasal Inspiratory Flow (PNIF) is another commonly used method for assessing nasal patency. PNIF provides an objective measurement of nasal airflow obstruction. It has the advantage of being simple, non-invasive and easily taught so participants can perform it on their own. The outcome is a direct representation of nasal congestion and therefore can provide objective confirmation of the subjective TNSS or VAS [26,37]. Repeating these measurements at intervals during the study can help identify the onset of action of medications.

The aim of the AR-CIC was to develop and optimize Standard Operating Procedures (SOPs) for each step of the NAC model, including participant eligibility, allergen introduction, symptom recording, and sample collection, through the pilot studies described here. This process allows multi-centre clinical trials to occur with more consistency and reliability.

## Methods

This study was conducted following approval from the Research Ethics Board of each site, and written informed consent was obtained from each participant.

### Inclusion and exclusion criteria

Inclusion criteria included a history of developing AR symptoms on exposure to an inhaled allergen to which the participant had a positive skin prick test (≥3 mm wheal compared to the negative control).

Participants were excluded if they had a history of a clinically significant chronic disease deemed limiting to tudy participation; a positive history for HIV, TB, HBV, or HCV; any nasal structural abnormalities or polyps; current signs/symptoms of active perennial rhinitis or seasonal AR; a history of frequent nasal bleeding or nasal surgery within the past 3 months; recent respiratory infection, or a history of smoking. Females who were pregnant or planned on becoming pregnant were excluded. A review of medical history and a nasal exam were performed on each visit.

Asthmatic patients were excluded from this pilot study, with the exception of exercise induced bronchospasm requiring occasional use (≤2 times per week) of a short acting beta agonist. A TNSS score of greater than 2 at screening, immediately before the challenge, was reason for exclusion since it indicated either AR from confounding allergies or non-specific hypersensitivity. Participants were instructed to refrain from taking allergy medications for the following periods of time before each study visit; 30 day washout period for systemic corticosteroids and astemizole; 14 days for inhaled/intranasal/intraophthalmic corticosteroids, cromolyn, leukotriene receptor antagonists, and beta blockers; 7 days for H_1_ and H_2_ receptor blockers; 3 days for anti-allergic ophthalmic treatments; 48 hrs for decongestants; 24 hrs for saline nasal sprays or ocular drops. Participants were also excluded if they were receiving allergen specific immunotherapy.

### Participants

Participants were recruited using multiple methods including searching a database of potential study volunteers, advertising, or posters displayed in allergy clinics. The Qualifying Allergen Concentration (QAC) study was conducted at 4 sites across Canada and were as follows from East to West: 5 participants at Université Laval (Quebec City, PQ), 10 at Queen’s University (Kingston, ON), 5 at McMaster University/St. Joseph’s Healthcare (Hamilton, ON), and 3 at the University of Alberta (Edmonton, AB). Two participants were excluded because of missing data on some of the symptom diary cards. An additional 4 people failed the screening process during the QAC study.

At all 4 sites, a total of 20 healthy, ambulatory, male and female participants between the ages of 18–65 years, completed the QAC study involving a single concentration of allergen during the NAC visit. A follow-up study using a Cumulative Allergen Concentration (CAC) was conducted at Queen’s University with 10 allergic and 4 non-allergic participants, while 6 people failed the screening process, due to not reaching the qualification criteria following administration of the highest allergen concentration.

### Study design

Each study consisted of 2 visits, a screening visit with a qualifying allergen challenge, and a NAC visit. The screening allergen challenge visit had to be conducted at least 7 days before the NAC visit. The allergen chosen for each participant was determined using clinical history and skin prick tests to a panel of common aeroallergens (ALK-Abelló, Denmark and HollisterStier Allergy, USA). At Queen’s University, all participants were challenged to ragweed while other sites recruited participants allergic to grass, house dust mite (*D. pteronyssinus* and *D. farinae*), cat dander, or ragweed. Spirometry was used to measure FEV_1_ (Forced Expiratory Volume in the first second) to confirm that it was safe for the participants to enrol in the study.

The qualifying participants were then challenged at screening using titrated allergen concentrations to determine their qualifying allergen challenge dose. An initial nasal wash with 5mls of 0.9% saline was used to identify and exclude participants with non-specific nasal hyper-responsiveness. Participants were asked to record their baseline symptoms and PNIF scores (In-Check; Clement Clarke International Ltd., Essex, UK). Individuals with a TNSS greater than 2 were excluded.

### Nasal allergen challenge

Allergen concentrations were prepared from stock solutions (ALK-Abello at Queen’s and McMaster sites, Hollister Stier at Laval and University of Alberta sites) in serial dilutions from 1:2048 to 1:2: each concentration was a 4-fold dilution of the previous. Starting with the most dilute dose (1:2048), 100 μL of the diluted allergen was sprayed into each nostril using the Aptar Bi-dose device (Aptar Pharma), and after 15 minutes PNIF and TNSS were recorded. If a PNIF reduction of ≥50% from baseline OR a TNSS score of ≥10/12 were achieved, the qualifying concentration to be used at the NAC visit was established. If the target values were not reached, the concentration incrementally increased until either the target PNIF or TNSS were achieved. If the participant reached the highest concentration of 1:2 without qualifying they were excluded.

At the NAC visit the qualifying allergen concentration from the screening visit was used as the single concentration for nasal challenge. PNIF and TNSS were recorded by the participants before the challenge, then at 15 min, 30 min, 1 hour, and then hourly for 12 hours following the nasal challenge.

The CAC study conducted at Queen’s University used a cumulative concentration of all the doses administered during the screening visit and this new allergen concentration was used at the NAC visit. For example, a participant who qualified at 1:128 at screening would receive a cumulative concentration of 1:98 (by adding the individual allergen concentrations of 1:2048, 1:512, and 1:128). The CAC also had a further modification in the qualifying criteria, in that participants qualified when they reached a PNIF fall of ≥50% AND a TNSS of ≥8/12.

### Symptom diary cards

Participants recorded their nasal symptoms on diary cards that included symptoms of runny nose, nasal congestion, sneezing and nasal itching. Each symptom was scored from 0–3, 0 indicating the absence of the symptom and 3 describing the symptom as severe and intolerable [6,38,39] (Table 1). The card was designed for automatic scanning and reading using Optical Mark Recognition (OMR) to allow automated data entry into the system.

### Statistical analysis

Graphpad Prism was used to generate the mean TNSS or PNIF and standard error of the mean at each time point of the NAC visits of the QAC and CAC studies. One way repeated measures ANOVA with Tukey multiple comparisons was used to calculate the significance of the mean at each time point by comparing all the time points to baseline and to the peak TNSS time point. The two-way repeated measures ANOVA with Bonferroni multiple comparisons was employed in assessing the different subsets of the QAC study. Correlation between the mean TNSS with PNIF over time was studied using Pearson’s Coefficient while Student’s t-test helped identify the statistical difference in TNSS at screening and NAC visits for both studies.

## Results

### Achieving the qualifying criteria in QAC study (screening visit)

Based on the qualifying criteria for the QAC study, 13 participants met the PNIF criteria of 50% fall without reaching a TNSS of 10/12, 4 participants met both criteria, and 3 achieved a TNSS of 10/12 only. The group that qualified based on “PNIF only” achieved a mean PNIF fall of 64%, which is significantly different (p < 0.001) from the “TNSS only” group who only scored a mean fall in PNIF of 27%. A difference was also noticed when comparing TNSS results where the “PNIF only” group reached a mean of 6.6 while the “TNSS only” group achieved a mean of 10.6 (p < 0.01). The group that achieved both qualifying criteria (PNIF fall of 50% AND a TNSS of 10/12 post NAC) was significantly different from the “TNSS only” group when comparing PNIF fall (p < 0.01) and different from the “PNIF only” group when comparing TNSS (p < 0.01). The “TNSS + PNIF” group achieved similar PNIF falls as the “PNIF only” group and similar TNSS scores to the “TNSS only” group, with no significant difference noted in these comparisons.

### Clinical responses at NAC during QAC study

**Figure 1:**
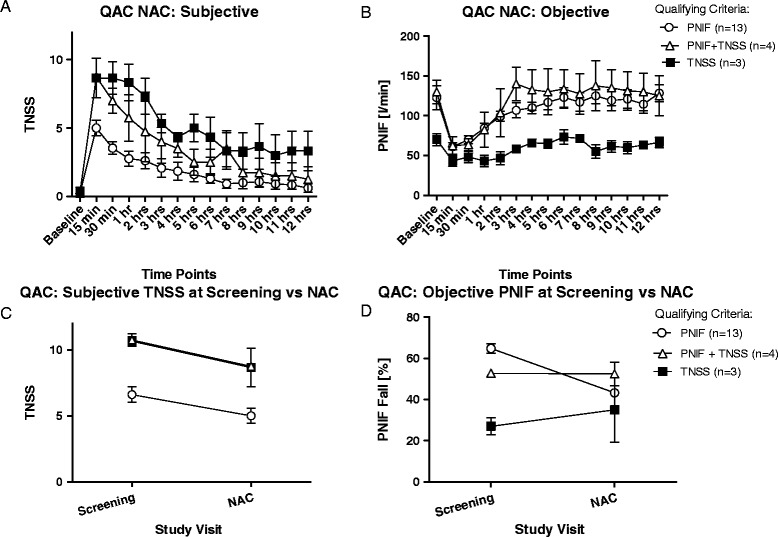
Analysis of QAC study. **A - B** show the mean and standard errors of TNSS and PNIF recorded by each group at each time point during the NAC visit (significance between time points: p<0.0001). The “PNIF only” group experienced consistently low TNSS over all time points (compared to “TNSS only” group: p<0.01 at 15 minutes and 1 hour, p<0.001 at 30 minutes; compared to “TNSS +PNIF” group: p<0.05 at 15 minutes) while the “TNSS only” group had the lowest PNIF recordings throughout the study. The “TNSS+PNIF” group had comparable TNSS to the “TNSS only” group and comparable PNIF to the “PNIF only” group. **C** - **D**. All 3 groups experienced a decline in TNSS from screening to NAC visit, but not all are statistically significant. “TNSS+PNIF” group had a significant (p<0.05) decline in NAC TNSS compared to screening. However, “TNSS only” and “TNSS+PNIF” groups both had a non-significant increase in PNIF during the NAC from screening while the “PNIF only” group experienced a decline (p<0.05).

The mean TNSS and PNIF are plotted for each time point for all 3 groups of participants in the QAC study during the NAC visit (Figure 1A-B). The 15 minute time point is consistently the peak TNSS or the lowest PNIF score for all 3 groups, after which there is a gradual drop in TNSS or a rise in actual PNIF compared to baseline measurements. The “TNSS only” group recorded the lowest PNIF measurements at all time points while the group that qualified using both criteria achieved similar PNIF measurements to that experienced by the “PNIF only” group (Figure 1B). There was no statistically significant difference between the groups when comparing PNIF scores. The “PNIF only” group experienced the lowest TNSS with significant difference (p < 0.05) at 15 minutes compared to the “TNSS + PNIF” group, and at 30 min. (p < 0.01), 1 hr (p < 0.001), and 2 hrs (p < 0.01) compared to the “TNSS only” group (Figure 1A).

A further comparison of the highest TNSS score at screening to the TNSS at 15 min. post NAC for each group showed that there is a drop in the score although only significant for “TNSS + PNIF” group (p = 0.0163) (Figure 1C). The same analysis of PNIF fall show that there is only a significant difference for the “PNIF only” group (p = 0.0307) between the screening and NAC visits, while the other groups maintained the same measurements achieved in the screening visit (Figure 1D).

### Seasonal vs Perennial allergens in QAC study

TNSS and PNIF scores for QAC participants were grouped according to the type of allergen they were allergic to (Table 2), whether seasonal or perennial, and then compared their screening scores to the NAC scores. 14 participants were challenged with seasonal allergens while 6 were challenged using perennial allergens. The Queen’s University site only included ragweed allergic (seasonal) participants while other sites chose allergen using the participant’s largest skin test result. Participants challenged with perennial allergens were more consistent in achieving the same TNSS scores during the NAC compared to what they reached in screening. Participants challenged with seasonal allergens experienced lower TNSS during the NAC compared to screening (p < 0.001). Making the same comparison using PNIF fall showed that both participant groups with seasonal and perennial allergies experienced a decrease in PNIF fall during the NAC but is not statistically significant.

**Table 2:** **Allergen used for each participant and the concentration at which they qualified during the screening visit**

**QAC Study**				**CAC Study**			
	**Study ID**	**Allergen**	**Max. Allergen Conc.**		**Study ID**	**Allergen**	**Max. Allergen Conc.**
Queen’s University	A046 109	Ragweed	1:128	Queen’s University	A083 117	Ragweed	1:8
	A017 110	Ragweed	1:128		A015 114	Ragweed	1:128
	A096 112	Ragweed	1:128		A053 118	Ragweed	1:32
	A050 113	Ragweed	1:128		A044 119	Ragweed	1:32
	A081 105	Ragweed	1:32		A079 116	Ragweed	1:8
	A033 104	Ragweed	1:512		A056 115	Ragweed	1:2
	A019 106	Ragweed	1:32		A016 125	Ragweed	1:32
	A001 108	Ragweed	1:2048		A060 124	Ragweed	1:128
	A066 103	Ragweed	1:128		A083 129	Ragweed	1:32
	A090 102	Ragweed	1:512		A048 131	Ragweed	1:32
Université Laval	A070 301	Std D. farinae	1:32		A002 123	Non-Allergic	1:2
	A095 302	Grass mix (5)	1:32		A061 126	Non-Allergic	1:2
	A008 303	Grass mix (5)	1:32		A088 127	Non-Allergic	1:2
	A038 304	D. pteronyssinus	1:32		A085 133	Non-Allergic	1:2
	A073 305	Std D. farinae	1:512				
McMaster University	AR001	D. pteronyssinus	1:32				
	AR002	Grass	1:2048				
	AR003	Cat hair	1:32				
University of Alberta	A275 404	Grass mix	1:2				
	A749 405	D. farinae	1:32				

Non-allergic participants in the CAC study did not meet the qualifying criteria even after challenge with the highest concentration (1:2). During the NAC visit, the 1:2 concentration was used once again.

### Outline of QAC and CAC study results

**Figure 2:**
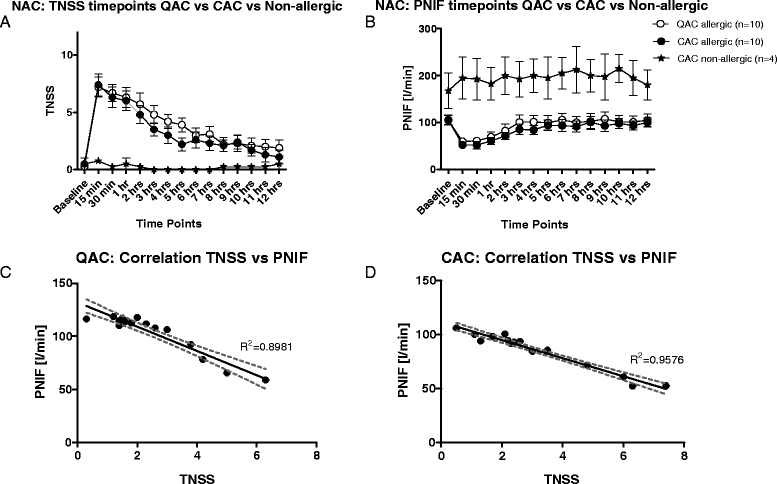
Comparing QAC (Queen’s University only) and CAC studies. **A** and **B**. Mean TNSS and PNIF scores including non-allergic participants. Allergic participants experienced the highest scores at 15 minutes (QAC TNSS: 15 minutes vs 4 and 5 hour time point p<0.05, vs 6^th^ hour to 12^th^ hour p<0.001) (CAC TNSS: 15 minutes vs 2 hour p<0.05, vs 3 to 12^th^ hour p<0.001) compared to non-allergic participants who did not experience change from baseline. Most time points were significantly greater than baseline scores (QAC: Baseline TNSS vs 15 minutes to 3 hours p< 0.001 and p<0.01 at the 4^th^ and 5^th^ hours) (CAC: Baseline TNSS vs 15 minutes to 2 hours p<0.001, vs 3^rd^ hour p<0.01, vs 4^th^ hour p<0.05). PNIF scores followed a similar pattern (QAC PNIF: Baseline vs 15 and 30 minutes p<0.001, vs 1 hour p<0.01; while 15 minutes time point vs 3 hours to the 12^th^ hour p<0.001) (CAC PNIF: Baseline time point vs 15 minutes up to 1 hour p<0.001 and vs 2 hours p<0.05; while the 15 minute time point vs 3 hours p<0.05, vs 5^th^ to the 7^th^ hour p<0.01, 8^th^ hour p<0.001, 9^th^ to the 11^th^ hour p<0.01, and 12^th^ hour p<0.001). While there were significant differences between the time points for allergic participants (two way ANOVA p<0.0001 for both TNSS and PNIF in both studies), there were no such significance experienced by non-allergic participants. **C** - **D**: Strong correlation between TNSS and PNIF exists in both studies, although the correlation is stronger in the CAC study (Person’s correlation QAC: R^2^=0.8981 p<0.0001, CAC: R^2^=9576 p<0.0001).

The mean TNSS for all allergic participants at each time point was plotted along with the standard error of the mean (Figure 2A). Data from both studies followed the same trend, with a peak in TNSS at 15 minutes post allergen challenge followed by a gradual decline. Statistical significance was achieved starting at 4 hours after allergen exposure onwards compared to the peak during the QAC study (15 min. vs 4 hrs, 5 hrs p < 0.05; 15 min. vs 6 hrs onwards p < 0.001) and starting from the 2 hour time point for the CAC study (15 min. vs 2 hrs p < 0.05; 15 min. vs 3 hrs onwards p < 0.001). While allergic participants in both studies had a significant change in TNSS from baseline measurements (p < 0.0001), non-allergic participants had no significant change during the 12 hours post exposure (p = 0.6044), suggesting the challenge did not cause nonspecific irritation (Figure 2A).

The mean PNIF at each time point also followed the same pattern as the TNSS, showing the greatest PNIF fall at 15 minutes post allergen challenge (QAC 15 min. vs 3 hrs and onwards p < 0.001; CAC 15 min. vs 3 hrs p < 0.05, 15 min. vs 5 hrs onwards p < 0.01) with a gradual increase of air flow thereafter (Figure 2B). The overall significance of the peak compared to baseline in both studies is p < 0.0001. In comparison, non-allergic participants did not experience any significant change in nasal air flow (p = 0.1323).

### Phenotypes of allergic rhinitis responses

**Figure 3:**
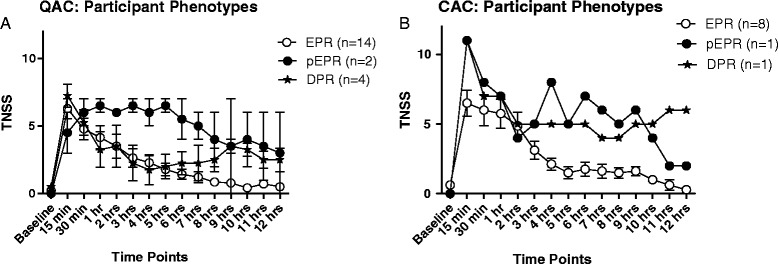
Participants divided based on the phases of AR experienced based on TNSS. Participants in the QAC (Figure 3 **A**) and CAC (Figure 3 **B**) studies who had an Early Phase Responders (EPR) and Dual Phase Responders (DPR) experienced a peak at 15 min. following NAC while those that experienced a protracted EPR (pEPR) had a gradual rise in TNSS up to 1 hr. This suggests that the 1hr time point ensures that the maximal amount of cytokines are secreted by this time point in all participants and therefore is the best time point for sampling nasal cytokines. While EPR experienced a drop in TNSS following the peak, pEPR maintained their symptoms level before declining, DPR experienced a drop in TNSS following the peak but did rise again later in the study. Statistical analysis was not possible since participant populations experiencing certain phases were too small to compare. Nevertheless, differences in scores over the 12 hour period were distinct from one participant population to the other.

For each of the studies, participants were separated into one of 3 groups, Early Phase Responders (EPR), protracted Early Phase Responders (pEPR), and Dual Phase Responders (DPR), depending on the TNSS pattern they experienced over the 12 hour period (Figure 3A-B). EPR had a decrease of ≥50% in TNSS by the 6^th^ or 7^th^ hour and did not experience another increase in TNSS but rather a return to baseline by the 12^th^ hour. DPR participants on the other hand had a sustained increase in TNSS beyond the 6^th^ or 7^th^ hour time point. A third group of participants, pEPR, who did not experience a 50% decrease in TNSS and did not return to baseline by the 12^th^ hour.

### Comparing protocols

Data collected from participants at Queen’s was used for this comparison since this was the only site that conducted both QAC and CAC studies, with 10 participants in each. In doing so, only participants allergic to ragweed were considered, eliminating any allergen-associated variability in nasal response. Although the CAC study achieved greater TNSS scores at screening, participants in both studies did not reach their qualifying scores when challenged at the NAC visit with the cumulative dose.

### Correlation between TNSS and PNIF

An inverse correlation exists between the subjective TNSS and the objective PNIF; as the mean PNIF decreases the mean TNSS increases (Pearson’s coefficient R^2^ = 0.8981 for QAC; R^2^ = 0.09576 for CAC) (Figure 2C,D).

### Adverse events

No adverse events were reported.

## Discussion

The pilot study helps fill a knowledge gap by evaluating the most appropriate methodology for nasal allergen challenge. We attempt to define what constitutes a positive response to allergen challenge, through modifying the qualifying criteria, and investigating the effects of allergen challenge given in a cumulative manner.

In this model, using simple inclusion criteria, such as history of AR and positive SPT to allergen was sufficient. From this pool, it is suggested that participants should be excluded on the basis of having active AR, any nasal structural abnormalities, and be able to observe washout periods for anti-inflammatory medications as well as not currently being treated with immunotherapy. From these pilot studies we can conclude that the CAC study had a better overall outcome for all participants by having a more robust qualifying criteria, among other changes.

Relying on either PNIF fall or TNSS alone as a measure of a positive challenge appears insufficient, since participants that met criteria for one of the measures did not necessarily meet the other. Both a minimum TNSS and a PNIF fall are needed to increase the chances of repeating the same scores in the NAC visit. The “TNSS + PNIF” group experienced significantly higher symptoms than either groups that qualified on only one criterion, similar to what was observed during screening.

Additionally, the “PNIF only” group did not manage to maintain the PNIF reduction, while the other groups did. The “TNSS + PNIF” group did not maintain their TNSS score which might be due to a short fall of the QAC protocol which uses a threshold dose allergen challenge protocol (as compared to the follow-up CAC study). It might also be the result of the limited number of participants that happened to qualify based on these criteria. Further evaluation of these qualifying criteria was needed and hence the CAC study was designed where all participants qualified based on both a fall in PNIF of 50% AND a TNSS of 8/12. The TNSS cut off was decreased from 10/12 to 8/12, as 10/12 was considered too challenging to achieve in general.

Participants in the QAC study qualified at lower concentrations than those in the CAC study (Table 2). This is almost certainly due to the more flexible qualifying criteria used in QAC (i.e. either a sufficient TNSS or PNIF fall halted the screening challenge) which resulted in participants qualifying earlier at a lower allergen concentration. In contrast, CAC participants qualified at higher concentrations as they were required to meet both the TNSS and PNIF criteria.

Participants challenged to a perennial allergen achieved comparable change in TNSS in the NAC as they did in the screening visit, unlike participants challenged with seasonal allergens. Perennial allergens are in the participants’ environment on a continuous basis and so they arrive to the study site “primed”, and more likely to achieve their earlier TNSS. An influx of inflammatory cells, such as eosinophils and basophils, during the late phase of AR, is responsible for such an effect [3]. It is recommended to have a one week interval between screening and NAC visits to help avoid such a priming effect. Ciprandi et al. studied the effects of nasal challenge performed at different intervals, evaluating both symptom scores and nasal cytology. The study concluded that a one week interval produced findings similar to the baseline challenge [40]. The use of non-standardized allergen extracts i is a potential limitation to our study, but to minimize challenges with recruitment, we chose to allow the use of non-standardized allergens if the skin test was positive to that extract and it was the most clinically relevant allergen for a given participant (e.g. ragweed or tree pollen).

When comparing PNIF fall data, participants challenged with perennial allergens had greater variability during the NAC, however, this was not significant. Shortfalls of using PNIF alone were explained earlier.

Using the combined criteria of both TNSS and PNIF, as subjective and objective measurements respectively, is an adequate way to effectively measure the progress of AR symptoms during direct NAC. Having a narrow score range to select from (0–3), participants generally do not avoid the extremes of the scale when assessing their TNSS [38,41]. Participants found the scale easy to use and did not require a significant amount of time to choose an adequate value to represent their symptoms and record it, especially during the short intervals between time points at the start of the NAC visit. Similarly, participants did not have any difficulty following instructions for measuring PNIF on their own, which is particularly important when considering recruiting large number of participants for phase II/III clinical trials or for studies requiring continued TNSS and PNIF recording while at home.

TNSS and PNIF fall for both studies demonstrated a strong correlation, indicating that using PNIF as an objective measurement is generally reliable in quantifying the allergic reaction. PNIF has been previously validated as an adequate representation of nasal signs, as found by Starling-Schwanz *et al.* who categorized participants based on anterior nasal signs observed via anterior rhinoscopy and correlated the results to PNIF [37]. Using PNIF and TNSS measurements to evaluate AR is therefore considered an accurate and simple way to obtain data at such frequent time points. Based on these measurements, the two studies demonstrated the effectiveness of allergen challenge via direct nasal spray as a valid method to challenge allergic participants.

The TNSS and PNIF pattern recorded during the NAC visit of the CAC study were similar to the “TNSS + PNIF” group in the QAC study, consistently reaching and maintaining higher TNSS and PNIF values. A subset of participants in both studies observed a decrease in TNSS and PNIF values during the NAC compared to the screening visit. Thus the change to the qualifying allergen dose ultimately administered appeared to accomplish more reliable results. Obtaining a robust response to allergen challenge is important to not only ensure one is including the most ‘allergic’ participants in a study, but in the context of a clinical trial one would have a greater symptom range to adequately assess the medication under investigation.

TNSS and fall in PNIF peaked at 15 minutes post NAC, which is consistent with previous studies of EPR (Figure 3A-B) [26]. Participants who experienced a pEPR in the QAC study reached their maximum symptoms later at 30 min. to 1 hr post NAC, which might suggest that the best time point to collect nasal samples for cytokine analysis is at 1 hr post NAC (Figure 3A) [42]. 75% of participants reached their peak TNSS by 15 minutes post-NAC, and 85% reached their peak by 1 hour. All allergic participants experienced the same consistent and repeatable trend in TNSS and PNIF, according to their respective group (EPR, pEPR, DPR), differing significantly from the non-allergic participants.

## Conclusions

Participants experienced reliable symptoms of AR in both studies and both dual responders and early phase responders were noted, an observation that resembles symptoms experienced by the general population [43,44]. This observation can be monitored in a study evaluating the efficacy of a novel medication over the full length of its duration of action [13,17,45]. Likewise, the NAC can be used to study how symptoms and cytokines are affected before and after immunotherapy as well as in drug safety studies [23,24]. The model is a safe method of studying medications in participants with asthma, since the technique ensures the allergen remains in the nasal cavity and is not inhaled into the lungs. Using the combined criteria of TNSS and PNIF fall is a more robust method of determining a positive challenge to nasal allergen administration.

Application of an optimized and standardized NAC model with improved SOPs are planned at multiple collaborating sites, allowing the AR-CIC to study AR pathophysiology and medication efficacy over a greater population nationwide. This collaboration, part of the AllerGen NCE, integrates the expertise of many researchers in future studies.
